# UNDERSTANDING THE NEEDS OF INFORMAL CAREGIVERS OF PEOPLE EXPERIENCING DEMENTIA AS IDENTIFIED ON SOCIAL MEDIA

**DOI:** 10.1093/geroni/igae098.1930

**Published:** 2024-12-31

**Authors:** Jane Flanagan, Maureen connolly, Amanada Coakley, Matthew (Brent) Fugate, Emily Prud’hommeaux, Colin Young

**Affiliations:** Boston College, Chestnut Hill, Massachusetts, United States; Boston College, Chestnut Hill, Massachusetts, United States; Endicott College, Beverly, Massachusetts, United States; Boston College, Chestnut Hill, Massachusetts, United States; Boston College, Chestnut Hill, Massachusetts, United States; Italina Home for Children, Boston, Massachusetts, United States

## Abstract

Social media has the potential to reach informal caregivers of people experiencing dementia and provide them with supportive and accurate strategies. This study aimed to understand the caregivers’ concerns posted on social media and determine if the responses were supportive and accurate. This study utilized a human-centered machine learning (HCML) framework, natural language processing (NLP), and information retrieval (IR) techniques to extract posts from a dementia caregiver group with the largest number of members (N = 59,954). Using a qualitative descriptive design, we thematically analyzed a sample of 500 initial posts. In a subset of 100 posts and responses to those posts, we used a Delphi method to determine if the responses were supportive and accurate. Five themes were identified: 1) Seeking information about unusual problem behaviors of dementia with nowhere to turn for help; 2) Grief, a sense of loss, and loneliness are profound; 3) Support from others is challenged by relationship issues and distance from caregiving; 4) Financial and legal issues present unexpected challenges; and 5) The need to celebrate caregiving is not recognized. The team found that 73% of the responses to initial posts were supportive, 10% were not, and 10%; did not address the concern; 73% of the responses were accurate, 1% were not and 10% did not address the concern. These findings highlight multifaceted concerns dementia caregivers face. The information is foundational to developing an interdisciplinary HCML interactive question-answering tool using NLP and IR to provide supportive and accurate information to dementia caregivers.

